# On the Anæmia of Infancy

**Published:** 1855-12

**Authors:** 

**Affiliations:** Vienna


On the Anæmia of Infancy. By Prof. Mauthner, of Vienna.—The author remarks, that for many years the practice of venesection has been on the decline, and he quotes the words of Professor Richter, of Dresden: "Poverty of blood is, next to tuberculosis and cancer, the increasing evil of our time, which will bring down a gradual deterioration of the race, and therefore merits our most earnest consideration." According to Valentiner, most neuralgic affections are caused by anæmia; and, according to Trousseau, chlorosis now prevails in the general pathology of the female. An anæmic mother will produce anæmic children; anæmia may be congenital, or acquired from too rapid development and quick growth. It is difficult to believe in the disease as congenital, the quantity of blood in the infant's tissues being normal. Valentin has shown that the amount of blood in the newly born is proportionately greater than in later life; for a child of five or six pounds has nearly two pounds of blood; while at the age of thirty it barely attains one-fifth of the weight of the body. But in infancy, as in old age, the watery constituent is more considerable than in middle life.

The cause of this congenital anæmia lies in the general corporeal weakness of the mother, whence also it comes that there are so many abortions and early deaths. Want of proper food during pregnancy exerts a potent influence, too commonly at work among the lower orders, oppressed with want and care; and the younger children are more subject to the disease, because the exhausted mother loses in time the power of nourishing her children by her own milk, and the father has not the means of procuring a wet nurse. A child born of a mother who has suckled another infant during her pregnancy, generally suffers from poverty of blood. Losses of blood, or profuse mucous discharges, by injuring the mother's health, are to be regarded as prejudicial to healthy fœtal development. An aged or diseased father commonly begets an anæmic child. Congenital syphilis must also be regarded as a cause. The morbid change in the blood being unknown, we must be content with the term "anæmia syphilitica." It is not always recognized, but it is of most momentous importance as regards the rising generation. Should an infant affected with this disease be vaccinated, a peculiar glandular disease is apt to ensue from this second poisoning of the blood. The author proceeds to enumerate the symptoms of congenital syphilis. The child suffers from excoriations about the mouth and anus, etc.; from roseola, pemphigus, eczema, psoriasis, or from a peculiar tenuity, smoothness, and transparency of the epidermis. They do not possess power to resist external influences; they are long in teething; tuberculosis is apt to ensue in the course of time.

The anæmia of development comes on when growth at any period is very quick. Hence we have the anæmia of dentition, the anæmia of puberty, or chlorosis.

From experiments upon animals, Nasse has shown that animal diet renders the blood more coagulable than vegetable diet, and increases the number of the blood corpuscles. From sugar and starch meal there is formed a glutinous lymph plasma, but no corpuscles. A purely vegetable diet, therefore, is not suited for infancy; the more so from the anatomical fact that the cæcum, that part of the intestinal canal where vegetable digestion goes on, is but imperfectly developed at that period. Anæmic children are very apt to suffer from inflammations. Nature endeavors to excite a reaction from this depressing influence; and stasis of the blood commonly ensues in organs unfitted for active circulation; but the exudations tend only yet more to impoverish the blood; and venesections materially increase the evil. One of the evils of infantile anæmia is hemorrhage from the congested and delicate vessels of the larger intestine. This occurrence is frequently overlooked, or confounded with other disorders, such as convulsions. The author has verified the fact by dissection.

When the plastic power of the organism, says Constatt, is exhausted by rapid natural and morbid evolutions, then tuberculosis suddenly forms, and the latent material begins to be deposited upon any excitement in the different viscera. Thus, it is to be feared, during the blood impoverishment of dentition; and it attacks especially children subject to perspirations, and to excited pulsations of the heart.

The practitioner should never forget, in considering the diseases of childhood, that sudden attacks, even death itself, may occur just as easily in children from anæmia as from hyperæmia. Thus, cases of convulsions and other attacks, once regarded as inflammatory, require, in the present day, more careful examination and more accurate diagnosis.—*Med. Times and Gaz.*

